# Varying models of intra-abdominal hypertension and their effect on renal function in a porcine model

**DOI:** 10.1186/cc12357

**Published:** 2013-03-19

**Authors:** R Wise, R Rodseth, I De Laet, L Luis, L Correa-Martin, M Garcia, G Castellanos, M Malbrain

**Affiliations:** 1 University of Kwazulu Natal, Durban, South Africa; 2 ZNA Stuivenberg, Antwerp, Belgium; 3 Neuron NPh, S.A., Granada, Spain; 4 Jesús Usón Minimally Invasive Surgery Centre, Cacères, Spain; 5 'Virgen de la Arrixaca' University Hospital, Murcia, Spain

## Introduction

Intra-abdominal hypertension (IAH) is an independent predictor of renal impairment and mortality [1]. Organ dysfunction caused by the pressure effect of IAH is well understood, but how this is modified in the presence of bowel obstruction is unclear. The aim of this study was to determine how different IAH models cause renal dysfunction in a pig model.

## Methods

Twenty-four pigs were divided into three groups; a control group (*n *= 5), a pneumoperitoneum (Pn) (*n *= 10), and an intestinal occlusion (Oc) model (*n *= 10). IAP was maintained for 3 hours at 20 mmHg during which time creatinine, urea, urine output, potassium, and glomerular filtration pressure (GFP) were measured. Statistical analysis was performed using repeated-measures ANOVA.

## Results

Over the first 3 hours there was a statistically significant difference between the control group and both IAH models for potassium (*P *= 0.002), urea (*P *= 0.045), creatinine (*P *= 0.012), GFP (*P *0.001), and urine output (*P *= 0.003). Over the full 5-hour period there was a statistically significant difference in the potassium measurement between the Pn and the Oc model (*P *= 0.01). The other parameters did not show a significant difference: urea (*P *= 0.171), creatinine (*P *= 0.074), GFP (*P *= 0.141), and urine output (*P *= 0.242).

## Conclusion

As expected the IAH models resulted in significantly worse renal function after 3 hours. This early renal dysfunction may be as a result of an early inflammatory process that has been associated with the pathophysiology of acute kidney injury. Potassium was significantly elevated in the Pn group as compared with the Oc group. Early changes in potassium levels with IAH may be a marker of early renal dysfunction and the usefulness of other renal biomarkers, such as NGAL, prompts further investigation.
